# The True and False Cundurango

**Published:** 1871-11

**Authors:** George J. Wenck

**Affiliations:** Chemist; 381 Sixth Avenue, N.Y.


### The True and the False Cundurango.

The favorable news which continue to come from the United States and other places of the virtues of this plant, renders it important to be able to distinguish the true Cundurango from other vines, which from a similar appearance are being substituted for it.

Dr. Chiriboga of Guayaquil, says, it is now fifteen months since I have made a particular study of the physical qualities and medical effects of the Cundurango, as well in the Hospital under my charge, as in private practice. I have observed attentively the action of this plant on various complaints, administered in the form of tincture, extract and decoction. I have proved to my own satisfaction that it has many admirable qualities, especially in the following :—

In Rheumatism (tendinous or muscular); in various Neuralgias; in Earaches and various skin diseases; in many diseases of the blood; in Cancerous diseases (saratanes), against which it may be looked on as a specific.

The astonishing improvement, obtained in two cases of open Cancer of the breast, and the complete disappearance of another non-ulcerated Cancer, as well as the marvellous curation of a cancer in the tongue of a gentleman who was once President of the Republic, and many other well attested cases, leave no room for doubt about the efficiency of the Cundurango in this terrible disease.

In the United States various publications have been made of the good effects of this plant in cancerous affections, even with the small quantity of Cundurango sent by the Government of Ecuador for that purpose; so much so, that speculations in the plant are now the rage, but unfortunately frauds have already been introduced and will tend to discredit this great discovery.

Whether from ignorance in some or cupidity in others, large quantities of a spurious species have been introduced into the market and particularly one, known by the name of *VejucoPachon*, to be exported as the true Cundurango, not being so in reality.

The former vine, it is true, belongs also to the family of *Asclepiaceæ*, being dicotyledenous gamopetalous, a climbing milky shrub, native of America. One of the number of the various species is cultivated in the gardens in Europe (Asclepiadea Syriaca) and known by the name of wild cotton or silk plant from the silky fibres, to which the seed are attached. The properties of this numerous family are so varied, that some are emetic, others tonic, anthelmintic, &c. It is only the Cundurango, which besides being tonic, deobstruent and diuretic, is distinguished by the singular and providential quality of being *anticancerous*.

Speculators in the plant ought to be careful to get the right species. Besides the botanical character, by which it is distinguished and the smallest quantity of the resin, peculiar to the Cundurango, evident at first sight, the following peculiarities may be useful to distinguish the two vines :—A concentrated decoction gives no reaction with test paper. That made with the *Vejuco Pachon* is slightly alkaline. That of the Cundurango, treated by ammonia gives a fine orange yellow tint, whereas the other yields a greenish yellow.

The Cundurango gives out an odor of pyroligneous acid treated by concentrated nitric acid, which is not the case in the other vine.

The decoction has a straw color and a characteristic odor, somewhat balsamic like nutmeg, which by itself distinguishes it from the other, which is turbid mucilagenous and inodorous.

There are two more species of Cundurango, one a pale yellow and the other black. The use of the latter requires much precaution in the doses given. The above characters are meant for the white Cundurango.

The bark is generally used, as the woody fibre is too weak.

For the information of the Medical profession the above observations, published in the "*Andes*," at Guayaquil, have been translated and are respectfully submitted.

GEORGE J. WENCK, *Chemist.*

381 Sixth Avenue, N.Y., November 1, 1871.

